# Recognition of Micro-Motion Jamming Based on Complex-Valued Convolutional Neural Network

**DOI:** 10.3390/s23031118

**Published:** 2023-01-18

**Authors:** Chongwei Shi, Qun Zhang, Tao Lin, Zhidong Liu, Shiliang Li

**Affiliations:** 1 Information and Navigation School, Air Force Engineering University, Xi’an 710077, China; 2 Equipment Management and Unmanned Aerial Vehicle Engineering School, Air Force Engineering University, Xi’an 710051, China

**Keywords:** inverse synthetic aperture radar (ISAR), micro-motion jamming, recognition, complex-valued convolutional neural network (CV-CNN)

## Abstract

Micro-motion jamming is a new jamming method to inverse synthetic aperture radar (ISAR) in recent years. Compared with traditional jamming methods, it is more flexible and controllable, and is a great threat to ISAR. The prerequisite of taking relevant anti-jamming measures is to recognize the patterns of micro-motion jamming. In this paper, a method of micro-motion jamming pattern recognition based on complex-valued convolutional neural network (CV-CNN) is proposed. The micro-motion jamming echo signals are serialized and input to the network, and the result of recognition is output. Compared with real-valued convolutional neural network (RV-CNN), it can be found that the proposed method has a higher recognition accuracy rate. Additionally, the recognition accuracy rate is analyzed with different signal-to-noise ratio (SNR) and number of training samples. Simulation results prove the effectiveness of the proposed recognition method.

## 1. Introduction

With the increasing complexity of electromagnetic environment, electronic warfare plays an important role in modern warfare [1]. Inverse synthetic aperture radar (ISAR) is widely used in modern radar warning systems (RWS), which provides high-resolution situation-awareness information for combat [2,3,4,5,6,7]. Noteworthy, the widely developed micro-motion feature extraction technology [8,9,10,11,12] makes it possible to realize a better target identification that is immune to the traditional ISAR jamming methods. ISAR jamming methods are mainly divided into suppression jamming [13,14] and deception jamming [15,16]. Suppression jamming can suppress the echo signal of the target by transmitting a high-power noise signal or various noise modulation signals. By modulating jamming signals containing the characteristic information of the real target, deception jamming can form false targets similar to the real target after ISAR pulse compression processing.

Recently, a new ISAR jamming method called micro-motion jamming was proposed. Different from the traditional ISAR jamming, the micro-Doppler effect generated by micro-motion modulation can affect ISAR imaging, making it difficult to identify the target correctly. Additionally, modulating jamming signals with the micro-motion feature of false targets can generate false micro-motion features. Thus, this kind of jamming method can work effectively against the micro-motion feature extraction technology and increase the difficulty of target identification [17,18,19,20]. Therefore, it is worthy to research an efficient way of anti-micro-motion jamming so that the ISAR can be well-performed. According to the operation principle, micro-motion jamming can be categorized into three types: modulation and repeater micro-motion jamming, micro-motion scattered wave jamming, and pulse convolution micro-motion jamming [21,22]. To ensure the effect of radar jamming, more than two jamming signals are simultaneously employed. Therefore, ISAR tends to be confronted with various jamming [23,24]. In this case, the recognition of ISAR jamming patterns is the prerequisite of taking corresponding anti-jamming measures. At present, related research has been carried out on the recognition of ISAR jamming patterns. The traditional research method of ISAR jamming recognition is to extract the signal features which can distinguish the target echo and different ISAR jamming patterns, then input the features into the classifier to realize ISAR jamming recognition [23,24,25,26]. Although these methods based on feature extraction can achieve a high recognition rate, it requires very professional and experienced man-rely features extraction ability to the determine recognition strategy. The efficiency of these methods may downgrade when various complex micro-motion jamming signals are simultaneously deployed.

Thanks to the development of artificial intelligence technology, deep learning has been successfully applied to ISAR jamming pattern recognition [27,28,29,30,31,32,33,34,35]. For instance, Wang et al. [27] implemented the recognition of jamming patterns by CNN for three kinds of jamming, including suppression jamming, multiple false targets jamming, and narrow-pulse jamming. The short-time Fourier transform image of the signal was used as input to CNN. Shao et al. [28] proposed a CNN-based Siamese network in order to solve the problem of insufficient training samples and recognized 12 kinds of radar jamming signals. Qu et al. [29] designed a jamming recognition network by integrating residual blocks and asymmetric convolution blocks with the power-spectrum characteristics of jamming signals, realizing the recognition of 10 kinds of suppression jamming signals. However, the jamming patterns recognized by these methods mainly focus on traditional suppression, and deception jamming signals. Research on pattern recognition for new types of jamming such as micro-motion jamming is still scarce and needs to be further investigated.

It should be noted that the micro-motion of the target will be directly attached in the radar echo, so most of the micro-motion features can be extracted by analyzing the complex echoes. Therefore, for micro-motion jamming signal recognition, this paper proposes a micro-motion jamming pattern recognition method based on complex-valued convolutional neural network (CV-CNN). In particular, micro-motion jamming is a kind of inter-pulse coherent jamming. It is difficult to identify micro-motion jamming patterns only from the signal features of a single pulse. Therefore, in this paper, multiple pulse signals of radar echoes are serialized and input to the network. The main contributions of this paper are as follows:

1. To our knowledge, this is the first time to realize micro-motion jamming recognition based on artificial intelligence technology. The simulation results verified the feasibility of well-performed classification ability.
2. Compared with methods based on real-valued convolutional neural network (RV-CNN), the proposed method can learn more detail features and thus improve the recognition accuracy effectively.
3. Compared to the traditional feature-based micro-motion jamming signal recognition methods, the proposed CV-CNN-based method can reduce the reliance of human-based feature extraction.

The additional contents of this paper are organized as follows. In Section 2, the mechanism of three typical kinds of micro-motion jamming patterns is introduced. In Section 3, the method of micro-motion jamming pattern recognition based on CV-CNN is given. In Section 4, relevant simulation results and comprehensive analysis are presented. In Section 5, conclusions of this paper are given.

## 2. Micro-Motion Jamming Signal Model

Assuming that ISAR transmits the linear frequency modulation (LFM) signal, it can be expressed as:

(1) $$s(\hat{t},t_{m})=\mathrm{rect}\left(\frac{\hat{t}}{\tau }\right)\cdot \exp\left[j2\pi \left(f_{0}t+\frac{1}{2}k{\hat{t}}^{2}\right)\right]$$

where τ is the pulse width, $f_{0}$ is the radar carrier frequency, k is the chirp rate, $\hat{t}$ is the fast time, $t_{m}$ is the slow time, and $t=\hat{t}+t_{m}$ represents the full time. rect(⋅) is the rectangular window function, which can be defined as:

(2) $$\mathrm{rect}\left(\frac{\hat{t}}{\tau }\right)=\left\{ \begin{array}{c} \;1\left|\hat{t}\right|\leq \tau /2\\ \;0\left|\hat{t}\right|\leq \tau /2 \end{array}\right.$$

### 2.1. Modulation and Repeater Micro-Motion Jamming

After intercepting the ISAR transmit signal, the jammer calculates the phase modulation of the false micro-motion point and the forwarding delay, and then forwards the jamming signal to the ISAR receiver after the phase modulation of the radar signal containing the micro-motion jamming information. Taking the rotating micro-motion point *P* for example, the phase modulation term of the jammer can be expressed as:

(3) $$e(t_{m})=\sigma_{P}\exp\left(-jr_{P}\sin\left(\omega_{P}t_{m}+\varphi \right)\right)$$

where $\sigma_{P}$ is the scattering coefficient of point *P*, $r_{P}$ is the rotation radius of point *P*, $\omega_{P}$ is rotation angular velocity of point *P*, φ is the initial phase. Without considering the noise, the radar echo of the point *P* can be described as:

(4) $$s_{P}(\hat{t},t_{m})=\mathrm{rect}\left(\frac{\hat{t}-2R_{P}(t_{m})/c}{\tau }\right)\cdot \exp\left[j2\pi \left(f_{0}\left(t-\frac{2R_{P}(t_{m})}{c}\right)+\frac{1}{2}k{\left(\hat{t}-\frac{2R_{P}(t_{m})}{c}\right)}^{2}\right)\right]$$

where $R_{P}$ represents the distance between the point *P* and the radar, c is the speed of light. Then the jamming signal after micro-motion modulation and forwarded by the jammer can be expressed as:

(5) $$\begin{array}{l} J_{1}\left(\hat{t},t_{m}\right)=s_{P}(\hat{t}-\Delta t,t_{m})\cdot e\left(t_{m}\right)\\ \;=\mathrm{rect}\left(\frac{\hat{t}-2R_{P}(t_{m})/c-\Delta t}{\tau }\right)\cdot e\left(t_{m}\right)\cdot \\ \;\exp\left[j2\pi \left(f_{0}\left(t-\frac{2R_{P}(t_{m})}{c}-\Delta t\right)+\frac{1}{2}k{\left(\hat{t}-\frac{2R_{P}(t_{m})}{c}-\Delta t\right)}^{2}\right)\right] \end{array}$$

where Δt represents the forwarding delay of the jammer. By changing the jammer forwarding delay and times, the location and number of jamming strips can be controlled to have different degrees of effect on ISAR imaging.

### 2.2. Micro-Motion Scattered Wave Jamming

After intercepting the ISAR transmit signal, the jammer modulates the micro-motion jamming information into the phase of the radar signal. Then, the jammer forwards the jamming signal to the target, which scatters the jamming signal to the ISAR receiver. Assuming that the distance between the radar and the micro-motion point *P* is $R_{RP}\left(t_{m}\right)$, and the distance between the jammer and the micro-motion point *P* is $R_{TP}\left(t_{m}\right)$, then the distance of the jamming signal echo path can be described as:

(6) $$R_{TPR}\left(t_{m}\right)=R_{RP}\left(t_{m}\right)+R_{TP}\left(t_{m}\right)$$

Therefore, the micro-motion scattered wave jamming signal can be expressed as:

(7) $$\begin{array}{l} J_{2}\left(\hat{t},t_{m}\right)=\mathrm{rect}\left(\frac{\hat{t}-2R_{TPR}\left(t_{m}\right)/c-\Delta t}{\tau }\right)\cdot e\left(t_{m}\right)\cdot \\ \;\exp\left[j2\pi \left(f_{0}\left(t-\frac{2R_{TPR}\left(t_{m}\right)}{c}-\Delta t\right)+\frac{1}{2}k{\left(\hat{t}-\frac{2R_{TPR}\left(t_{m}\right)}{c}-\Delta t\right)}^{2}\right)\right] \end{array}$$

The jammer and ISAR can be equivalently regarded as the transmitter and receiver of the bistatic radar. Similar to the modulation and repeater micro-motion jamming, the micro-motion scattered wave jamming can generate jamming strips in azimuth. In addition, supposing that the equivalent bistatic angle is α, according to the characteristic of scatter-wave signal, micro-motion scattered wave jamming can generate 2-D images of false targets similar to the target, which differ from the actual target image by the angle of α/2.

### 2.3. Pulse Convolution Micro-Motion Jamming

After intercepting the ISAR transmit signal, the jammer uses pulse sequences with different delays to convolve with it and then forwards the jamming signal to the ISAR receiver. This jamming pattern is essentially a time-delayed forwarding of micro-motion points within selected range cells. The false micro-motion point echoes are generated by controlling the forwarding delay parameters through looping in a fixed period. Assuming a micro-motion point *P* on the target, its radar echoes can be expressed as:

(8) $$s_{P}(\hat{t},t_{m})=\mathrm{rect}\left(\frac{\hat{t}-2R_{P}(t_{m})/c}{\tau }\right)\cdot \exp\left[j2\pi \left(f_{0}\left(t-\frac{2R_{P}(t_{m})}{c}\right)+\frac{1}{2}k{\left(\hat{t}-\frac{2R_{P}(t_{m})}{c}\right)}^{2}\right)\right]$$

Assuming that the jammer forwarding delay Δt varies cyclically with slow time $t_{m}$ within the interval $\left[t_{1},t_{2}\right]$, the pulse sequence can be described as:

(9) $$p\left(\hat{t},t_{m}\right)=\delta \left(\hat{t}-\Delta t\left(t_{m}\right)\right)$$

where δ(⋅) represents the unit impulse function. Specify $f\left(t\right)=f_{1}\left(t\right)*f_{2}\left(t\right)$ is the convolution of $f_{1}\left(t\right)$ and $f_{2}\left(t\right)$, the pulse convolution micro-motion jamming signal can be expressed as:

(10) $$\begin{array}{l} J_{3}\left(\hat{t},t_{m}\right)=p\left(\hat{t},t_{m}\right)*s_{P}(\hat{t},t_{m})\\ \;=\mathrm{rect}\left(\frac{\hat{t}-2R_{P}(t_{m})/c-\Delta t\left(t_{m}\right)}{\tau }\right)\cdot \\ \;\exp\left[j2\pi \left(f_{0}\left(t-\frac{2R_{P}(t_{m})}{c}-\Delta t\left(t_{m}\right)\right)+\frac{1}{2}k{\left(\hat{t}-\frac{2R_{P}(t_{m})}{c}-\Delta t\left(t_{m}\right)\right)}^{2}\right)\right] \end{array}$$

## 3. Recognition of Micro-Motion Jamming Based on CV-CNN

In this section, the main structure of CV-CNN is introduced, and the function of each part is analyzed. In addition, the processing process is described with the pre-processing of the input signal for the micro-motion jamming recognition.

### 3.1. The Structure of CV-CNN

**Figure 1:**
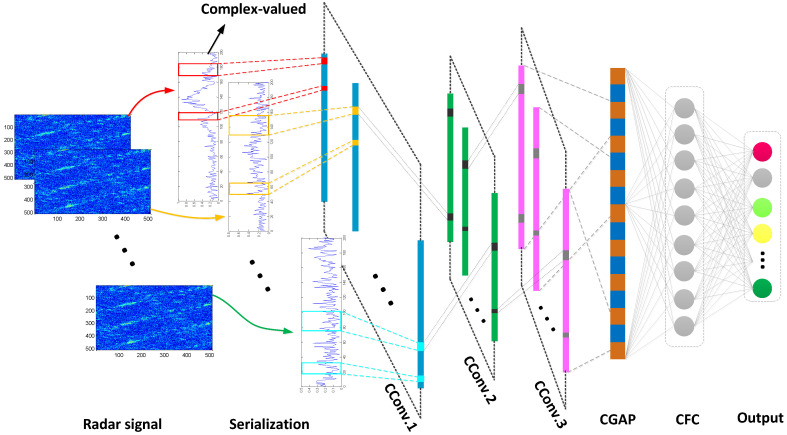
CV-CNN structure framework.

Considering that the recognized micro-motion jamming signals are complex-valued signals, CV-CNN can effectively preserve the amplitude and phase characteristics of the signal compared with RV-CNN. Similar to RV-CNN, CV-CNN consists of several cascaded layers, including an input layer, several convolution layers (including activation layers, pooling layers and dropout layers), a normalization layer, a global average pooling layer, a fully connected layer, and a Softmax layer. Meanwhile, a 1-D CV-CNN structure with serialization of 2-D multi-pulse signals is designed for efficient processing of 1-D signals, which will be explained in detail later. The proposed network framework of CV-CNN for micro-motion jamming recognition is shown in Figure 1.

In the complex convolution calculation, suppose that the *k*th output of the *l*-1th complex convolution layer is $a_{k}^{l-1}\in {\mathbb{C}}^{W_{l-1}\times H_{l-1}\times I_{l-1}}$, which is also the *k*th input of the *l*th convolution layer. Then, the *i*th output of the *l*th convolution layer is $a_{i}^{l}\in {\mathbb{C}}^{W_{l}\times H_{l}\times I_{l}}$ after convolution calculation, where ℂ denotes the complex domain. The convolution calculation of the *l*th convolution layer contains several complex convolution kernels $w_{ik}^{l}\in {\mathbb{C}}^{F_{l}\times F_{l}\times I_{l-1}\times I_{l}}$ and a bias $b_{i}^{l}$ [36]. Assuming that $A_{i}^{l}$ represents the result of the complex convolution calculation of the input $a_{k}^{l-1}$ and the complex convolution kernel $w_{ik}^{l}$, the calculation process can be expressed as follows:

(11) Ail=∑k=1Kwikl∗akl−1+bil    =∑k=1K(Re(wikl)∗Re(akl−1)−Im(wikl)∗Im(akl−1)+Re(bil))    +i∑k=1K(Re(wikl)∗Im(akl−1)−Im(wikl)∗Re(akl−1)+Im(bil))

where character ∗ represents the convolution calculation, Re(⋅) and Im(⋅) are the real and imaginary parts of the extracted complex values respectively. Then $a_{i}^{l}$ can be expressed as:

(12) ail=f(Re(Ail))+i⋅f(Im(Ail))

where f(⋅) denotes the nonlinear activation function. In the designed CV-CNN, we use the complex activation function modReLU, which is obtained by extending the ReLU activation function used for RV-CNN. The modReLU activation function can be expressed as:

(13) modReLU(a)=ReLU(|a|+b)eiθa={(|a|+b)a|a| , (|a|+b)≥00      , otherwise

where a is the input, $\theta_{a}$ is the phase of a, and *b* is the learnable parameter. By setting *b*, the activation function can reach the position of the dead zone since it is always positive. The complex data does not change its phase before and after passing through the activation function, which is the characteristic of modReLU activation function.

After the complex convolution calculation, the corresponding output is fed into the global average pooling layer. This layer is also extended from the real domain to the complex domain. Mathematically, the complex domain extension of the global average pooling layer is defined as:

(14) CGAP=G(Re(a))+i⋅G(Im(a))

where G(⋅) represents the global average pooling calculation in real domain.

Then, the output of the complex global average pooling layer is used as the input of the complex fully connected layer. After the calculation of the fully connected layer, the output complex result is taken as the input of the Softmax classifier after taking the magnitude to obtain the final recognition result.

The CV-CNN also uses a backpropagation algorithm in the process of updating the parameters. In this paper, the polynomial maximum likelihood function is used as the loss function. The values of weights and deviations can be updated by the following equations:

(15) $$w_{ik}^{l}\left(t+1\right)=w_{ik}^{l}\left(t\right)+\Delta w_{ik}^{l}\left(t\right)=w_{ik}^{l}\left(t\right)-\eta \frac{\partial L}{\partial w_{ik}^{l}\left(t\right)}$$

(16) $$b_{i}^{l}\left(t+1\right)=b_{i}^{l}\left(t\right)+\Delta b_{i}^{l}\left(t\right)=b_{i}^{l}\left(t\right)-\eta \frac{\partial L}{\partial b_{i}^{l}\left(t\right)}$$

The key lies in the calculation of the error gradient of the weights, which can be derived from the following equation:

(17) ∂L∂wikl=∂L∂Re(wikl)+i∂L∂Im(wikl)  =(∂L∂Re(Ail)∂Re(Ail)∂Re(wikl+1)+∂L∂Im(Ail)∂Im(Ail)∂Re(wikl))  +i(∂L∂Re(Ail)∂Re(Ail)∂Im(wikl)+∂L∂Im(Ail)∂Im(Ail)∂Im(wikl))

Suppose there is an intermediate quantity $\delta_{i}^{l}$ to represent the error term, whose mathematical can be expressed as:

(18) δil=−∂L∂Re(Ail)−i∂L∂Im(Ail)

According to Equations (11), (12), and (18), Equation (17) can be simplified as:

(19) $$\frac{\partial L}{\partial w_{ik}^{l}}=-\delta_{i}^{l}{\left(a_{i}^{l-1}\right)}^{*}$$

where ${\left(\cdot \right)}^{*}$ denotes taking the conjugate. Substituting the obtained gradient into Equation (15) to complete the update of the weight parameter. By iterating continuously, the error can be continuously reduced until it is minimized.

### 3.2. Signal Data Pre-Processing

Due to the specificity of convolutional calculation in CV-CNN, the convolution layer can be transformed into generalized matrix multiplication. In this case the data needs to be pre-processed before the network training is performed to accommodate a more efficient convolutional calculation.

**Figure 2:**
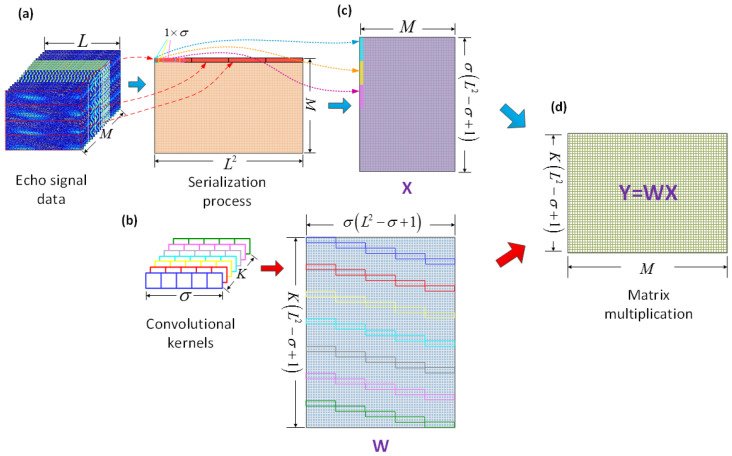
Standard procedure for generalized matrix multiplication of convolution layers. (**a**) Serialization process of echo signal data, (**b**) Expand convolutional kernels to form convolutional kernel matrix, (**c**) Form input matrix, (**d**) Obtain output matrix.

As shown in Figure 2a, the echo signal data of size L×L is converted into size $1\times L^{2}$ by serialization process. Assuming a total of *M* samples in the training set, which are combined into a complete 2-D data matrix for the training matrix of size $M\times L^{2}$. At the same time, the class-label matrix of the same dimension size is designed and stored. Then, the training matrix and the convolutional kernels are processed separately. We take *K* convolutional kernels with the size of 1×σ, and the step of 1 as an example. As shown in Figure 2b, all the convolutional kernels are expanded to form the convolutional kernel matrix *W* with size $K\left(L^{2}-\sigma +1\right)\times \sigma \left(L^{2}-\sigma +1\right)$. Meanwhile, the training matrix is patched according to the range of data covered by the convolutional kernels and the way of movement to form the input matrix *X* with size $\sigma \left(L^{2}-\sigma +1\right)\times M$, as shown in Figure 2c. Thus, it can match the convolutional matrix in size. As shown in Figure 2d, after the matrix operation, the output matrix Y=WX with size $K\left(L^{2}-\sigma +1\right)\times M$ can be obtained to complete the convolution calculation. After the matrix *Y* passes through the activation layer, it is used as the input data of the next convolution layer for a new round of calculation.

## 4. Simulations and Results

### 4.1. Datasets Design

**Figure 3:**
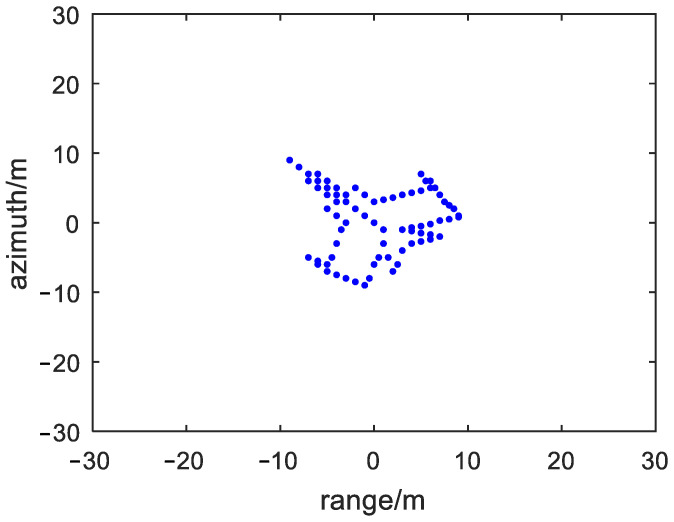
The aircraft scattering point target.

Assuming the ISAR transmit LFM signal, the carrier frequency $f_{0}$ is 10 GHz, the bandwidth B is 400 MHz, the pulse width τ is 5 µs, the pulse repetition frequency (PRF) is 200 MHz, and the pulse number is 512. The ISAR transmits signals to an aircraft scattering point target as shown in Figure 3, using ISAR rotation target imaging model. The rotation angular velocity of the target is set to 0.02 rad/s.

**Figure 4:**
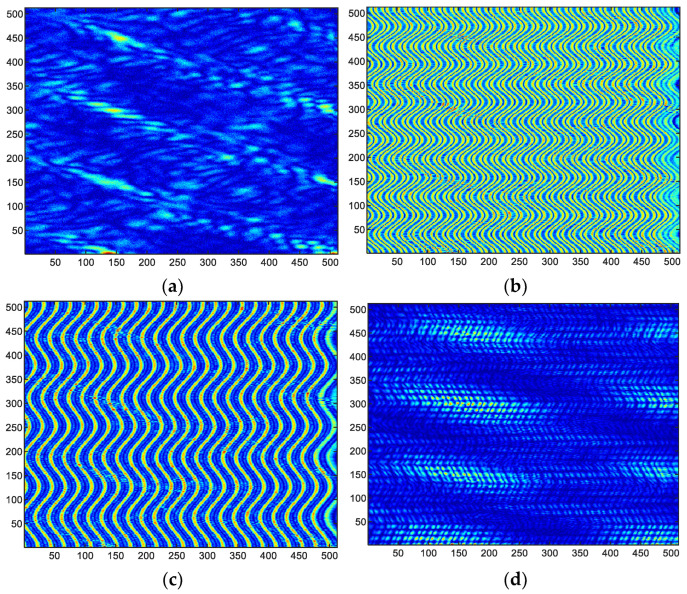
The images of four different classes of echo signal matrix. (**a**) Class1, (**b**) Class2, (**c**) Class3, (**d**) Class4.

According to whether the ISAR is jammed and different patterns of micro-motion jamming, the echo signals are divided into four classes as follows:

(20) $$\left\{ \begin{array}{l} \mathrm{Class}1:s\left(\hat{t},t_{m}\right)=s_{0}\left(\hat{t},t_{m}\right)+n\left(t\right)\\ \mathrm{Class}2:s\left(\hat{t},t_{m}\right)=s_{0}\left(\hat{t},t_{m}\right)+J_{1}\left(\hat{t},t_{m}\right)+n\left(t\right)\\ \mathrm{Class}3:s\left(\hat{t},t_{m}\right)=s_{0}\left(\hat{t},t_{m}\right)+J_{2}\left(\hat{t},t_{m}\right)+n\left(t\right)\\ \mathrm{Class}4:s\left(\hat{t},t_{m}\right)=s_{0}\left(\hat{t},t_{m}\right)+J_{3}\left(\hat{t},t_{m}\right)+n\left(t\right) \end{array}\right.$$

where $s_{0}\left(\hat{t},t_{m}\right)$ is the real target echo signal, $J_{1}\left(\hat{t},t_{m}\right)$ is the modulation and repeater micro-motion jamming signal, $J_{2}\left(\hat{t},t_{m}\right)$ is the micro-motion scattered wave jamming signal, $J_{3}\left(\hat{t},t_{m}\right)$ is the pulse convolution micro-motion jamming signal, and n(t) is additive white Gaussian noise. In order to display different complex-valued echo initial signals in the form of images, we take the complex values of the echo signal matrix as modulo values to generate images of the four echo signal classes as shown in Figure 4.

In the designed dataset, four classes of echo signal matrix are constructed as shown in Figure 4, which are target echo without jamming (Class1), modulation and repeater micro-motion jamming (Class2), micro-motion scattered wave jamming (Class3), and pulse convolution micro-motion jamming (Class4). Especially, the jamming parameters of the abovementioned three micro-motion jamming patterns are shown in Table 1.

10 groups of jamming parameters are selected within the range of jamming parameters shown in Table 1, and the parameters are chosen as randomly dispersed as possible while having good jamming effects. Three occasions with different signal-to-noise ratio (SNR) of 5, 0, and -5 dB are constructed. For each occasion, the samples of each class are randomly generated within the range of the jamming-to-signal ratio (JSR) of -10~20 dB based on Monte Carlo method. Among the samples, 500 samples per class are selected as the training set, 200 samples per class are selected as the validation set, and 150 samples per class are selected as the test set. Overall, there are four classes of echo signals (they are target echo signals and three micro-motion jamming signals) in the dataset, with 2000 training samples, 800 validation samples, and 600 test samples under each SNR. Each sample is a 512 × 512 matrix of a total of 262144 complex values.

### 4.2. Simulation Settings

In the simulation experiments, RV-CNN is used as a comparison in order to verify the effectiveness of the micro-motion jamming recognition method based on CV-CNN. The main sections of the network structure of RV-CNN and the parameter settings are shown in Table 2.

The RV-CNN for comparison consists of three convolution layers, three pooling layers, three activation layers, a dropout layer, a normalization layer, a global pooling layer, a fully connected layer, and an output layer. The output from the full connection layer is 4, which means it would support 4 properties for the four classes. The step lengths of the convolution layers are set to 1, and the ReLU activation function is used for the activation function.

The network structure and parameter settings for the proposed CV-CNN are shown in Table 3. It can be seen that the overall structure is similar to that from RV-CNN. The main difference is that CV-CNN uses modReLU activation function as the activation function, which is extended to complex domain compared with ReLU. It should be noted that the pre-processing for dataset of the CV-CNN reserves the complex data structure, while that of RV-CNN transfers the complex value into real value and extracts out the exact real data. For example, the complex value can be transferred to one real value by absolution, or it can divide the real part and the image part to be two real values.

The same hyperparameter settings are used for training with RV-CNN and CV-CNN, the range of learning rate is set to [0.00005, 0.005], the weight decay rate is set to 0.0001, batch size is set to 20, and the training epochs is set to 25.

### 4.3. Recognition Results and Analysis

**Figure 5:**
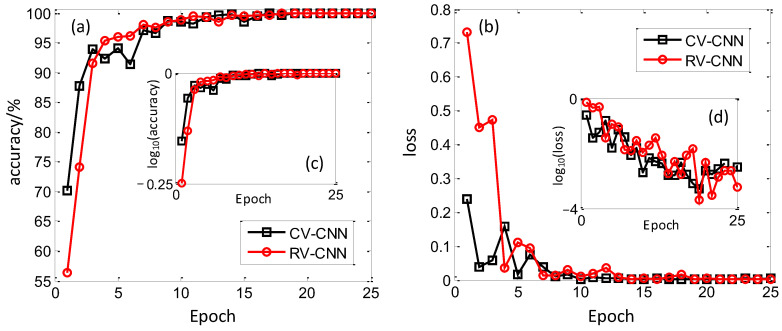
Training results of CV-CNN and RV-CNN on training set. (**a**) Training accuracy, (**b**) Training loss, (**c**) Training accuracy with logarithmic scale, (**d**) Training loss with logarithmic scale.

In this section, the dataset under SNR = −5 dB condition is analyzed, and the recognition results of CV-CNN and RV-CNN are compared. The training results are shown in Figure 5, and Figure 5a,b represent the training accuracy and training loss with increasing epoch for CV-CNN and RV-CNN, respectively. It can be seen that the training loss of both models gradually decreases and converges to a stable level as the training time grows. Among them, the training accuracy of RV-CNN starts from 56.33 to 100% and reaches more than 95% at epoch = 4. Its training loss function starts from 0.733084 to 0.000648. In contrast, the initial training accuracy of CV-CNN is 70.07% and converges to about 95% at epoch = 3, with an initial training loss function of 0.239535; additionally, its convergence speed is much faster. To better merge the training and validation results, we introduce the logarithmic function to discern the variation. As shown in Figure 5c,d, the logarithmic processing is not very apparent for training accuracy because the values are adjacent to 1. Fortunately, it is easily observed for training loss, and the obvious decreasing tendency can be seen. For CV-CNN and RV-CNN, the training loss changes with four orders, which exemplifies the excellent training ability of these models.

**Figure 6:**
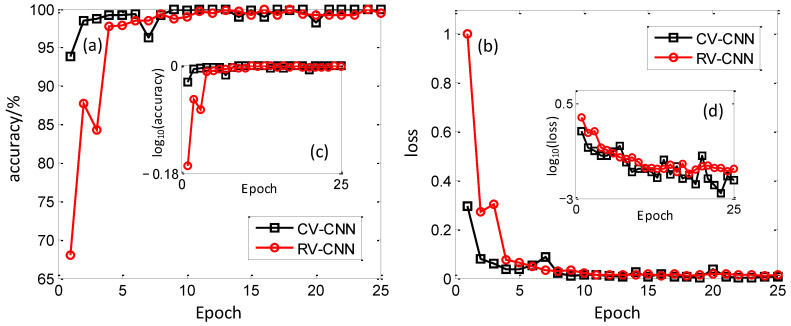
Validation results of CV-CNN and RV-CNN on validation set. (**a**) Validation accuracy, (**b**) Validation loss, (**c**) Validation accuracy with logarithmic scale, (**d**) Validation loss with logarithmic scale.

After that, the trained model is adjusted by using the validation set to improve the generalization ability of the model, and the results are shown in Figure 6. It can be seen that the hyperparameter validity of CV-CNN is significantly higher than that of RV-CNN, both in terms of validation accuracy and validation loss; where CV-CNN has an initial value of 93.8% validation accuracy and has reached about 95% accuracy at epoch = 2, while RV-CNN has an initial value of 68% accuracy and reaches the same accuracy only at epoch = 4. In terms of validation loss, the initial loss value of CV-CNN is much lower than that of RV-CNN, and the loss value is already less than 0.1 at epoch = 2. Overall, the learning ability of CV-CNN is better than that of RV-CNN, because CV-CNN extracts more refined signal features and can train the model to show better results more rapidly. Similarly, the validation results are also logarithmically processed to achieve a better presentation effect. As can be seen in Figure 6c,d, the improvement for validation accuracy is not ideal, but for the validation loss, it is worth trying. In Figure 6d, near three orders variation can be observed thanks to the good training ability of these models.

**Figure 7:**
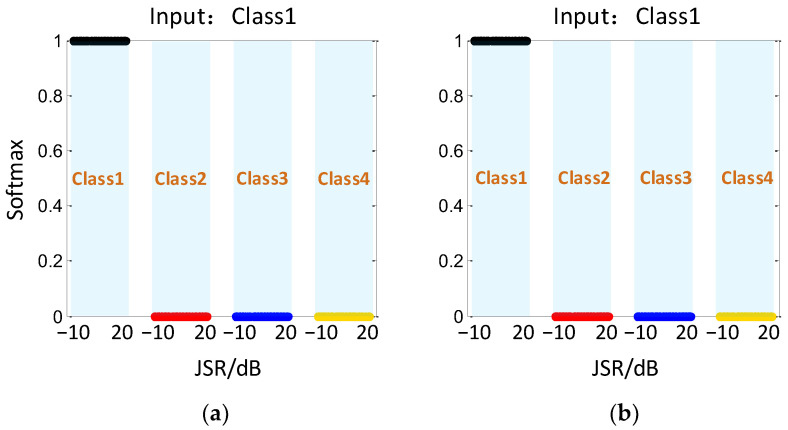
Recognition results with different JSRs when the input is Class1. (**a**) CV-CNN, (**b**) RV-CNN.

Finally, a test set is used to test the CV-CNN model and RV-CNN model in recognizing the four echo signal classes. The test assigns 150 samples to each class, and the JSR of the samples is randomly distributed in the range of −10 dB~20 dB. Figure 7 shows the recognition output results based on CV-CNN and RV-CNN when the input is the target echo without jamming (Class1). It can be seen that both models have the best recognition ability for Class1 with no error output.

**Figure 8:**
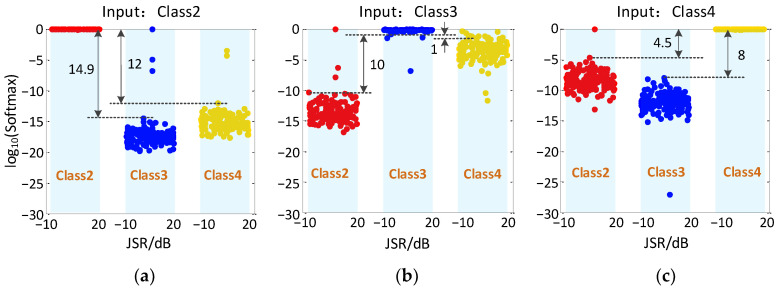
Recognition results of CV-CNN with different inputs and different JSRs. (**a**) Input is Class2, (**b**) Input is Class3, (**c**) Input is Class4.

**Figure 9:**
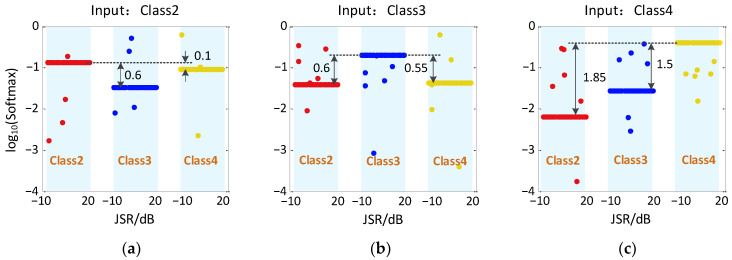
Recognition results of RV-CNN with different inputs and different JSRs. (**a**) Input is Class2, (**b**) Input is Class3, (**c**) Input is Class4.

It should be noted that in the process of recognition for Class1, the Softmax function outputs recognition probability results of only 1 and 0 due to the good recognition effect, so the original results can be shown in the image directly. However, for Class2~Class4, they have probabilistic results with recognition rates of 10^−3^ or less, which are difficult to display directly in the image. Therefore, in the following analysis, log_10_(Softmax) is used to represent the output results to show more intuitively the distribution range of different output results. In particular, since the signal characteristic of Class1 is much more significant than other jamming signals, the probability of Class2~Class4 being misclassified as Class1 in the model output is 0. Because of log_10_(0) = −∞, the output results cannot be displayed in the image, thus only the recognition results of Class2~Class4 are shown in Figure 8 and Figure 9.

Figure 8 shows the output results of CV-CNN model for Class2~Class4 on a test set. It can be seen that when the input is Class2, most of the samples are recognized as Class2, with only one sample misclassified as Class3. When the input is Class3, two samples are misclassified as Class2, and thhree samples are misclassified as Class4. When the input is Class4, only one sample is misclassified as Class2, and the rest are correctly recognized.

Figure 9 shows the output results of RV-CNN model for Class2~Class4 on a test set. It can be seen that when the input is Class2, two samples are misclassified as Class3 and one sample is misclassified as Class4. When the input is Class3, two samples are misclassified as Class2 and four samples are misclassified as Class4. When the input is Class4, one sample is misclassified as Class2, two samples are misclassified as Class3, and the rest are correctly identified.

By comparing the results in Figure 8 and Figure 9, it can be found that the distribution of recognition probability points of the CV-CNN model is variously scattered, while the results from RV-CNN model are more stable and keep in exact level. The reason is that the CV-CNN extracts more signal features and can describe the signal nuances. To better measure the recognition ability of the two models for the micro-motion jamming signals recognition, the concept of probability distance is introduced as follows:

(21) $$P_{\mathrm{XY}}=\log_{10}\left(\frac{P_{prc\_true\_\mathrm{ClassX}}}{P_{conf\_\mathrm{ClassY}}}\right),\;X,Y=1,2,3,4$$

where, $P_{prc\_true\_\mathrm{ClassX}}$ represents the probability that ClassX is correctly classified and $P_{conf\_\mathrm{ClassY}}$ represents the probability that ClassX is misclassified as ClassY. A larger probability distance means the recognition ability is better and less likely to confuse the signals. The probability distance based on CV-CNN in Figure 8 is up to 14.9, but the probability distance based on RV-CNN in Figure 9 is only up to 1.85. In comparison, RV-CNN is more likely to confuse the signals.

**Figure 10:**
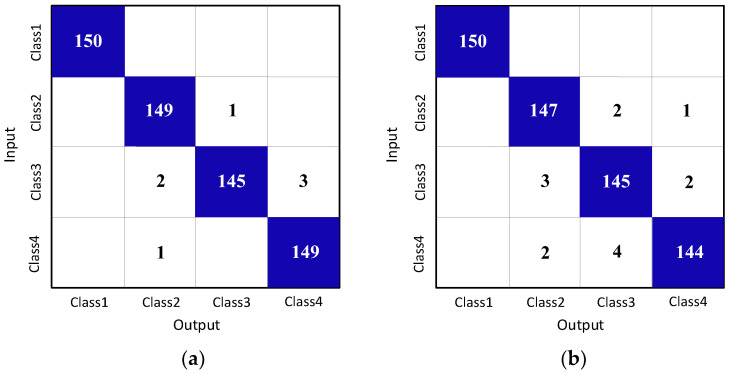
Confusion matrix results of CV-CNN and RV-CNN on test set. (**a**) CV-CNN, (**b**) RV-CNN.

According to the above results, the confusion matrix is shown in Figure 10, where Figure 10a is the result of CV-CNN and Figure 10b is the result of RV-CNN. In comparison, CV-CNN has better recognition ability of micro-motion jamming.

Based on the above recognition results, the comparison of the recognition ability of CV-CNN and RV-CNN is summarized in Table 4. It can be seen that the recognition ability of CV-CNN for micro-motion jamming signals is better than that of RV-CNN. The reason is that training in the complex field can effectively retain more signal features and have better recognition ability.

In addition, the construction and division of the dataset often affects the training effect of the model. The effects of different SNRs and training set sizes on the training effect of CV-CNN model will be studied below. The training sets with SNR of −5 dB, 0 dB, and 5 dB are selected, and the training set sample size of various signal patterns is set to 50, 100, 150, 200, 250, and 300. Moreover, four kinds of signals are mixed in the test dataset. Here, we introduce an overall accuracy to evaluate the effectiveness of the method. It can be expressed as a rate between right classified sample number *n* and the total sample number *N*. The training and test results are shown in Table 5.

As can be seen from the table, the overall recognition accuracy of the model improves significantly as the SNR increases for the same training samples. In addition, increasing the number of samples in the training set enables the model to learn more detail features of the signals. Therefore, as the number of the samples increases, the recognition accuracy also improves under the same SNR.

## 5. Discussion

**Figure 11:**
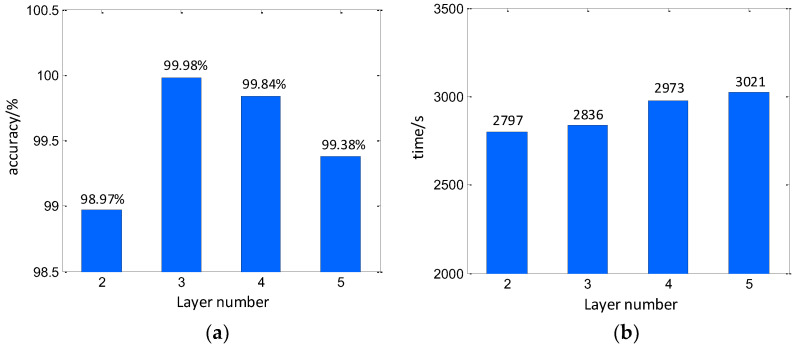
Training results under different number of convolutional layers. (**a**) Accuracy, (**b**) Time.

In discussion, the hyperparameters are mainly considered. First, the convolution layer number is a significant parameter that determines the structure of the CV-CNN. In this work, the layer number is investigated to find the optimal convolution size. In this investigation, the convolution structures with the layer numbers of 2, 3, 4, and 5 are constructed. The kernel numbers are set to (32, 64), (32, 64, 128), (32, 64, 128, 256), and (32, 64, 128, 256, 512). Noteworthy, larger layer numbers require more computer memory and should be occupied to perform the training. It may downgrade the efficiency. However, a simplified convolution structure can hardly describe the exact signal figuration. Based on the parameter setting above, the experiment is carried out. The overall classification accuracy for different layer numbers is shown in Figure 11. It can be seen that, when considering the accuracy, larger layer number would provide a better classification result. However, for the time cost, lower layer numbers can be more efficient.

Besides the convolution layer number, other hyperparameters such as the learning rate, batch size, and number of convolutional kernels also make significant contributions to the deep learning results. Moreover, artificial intervention is required. In this part, a dataset with SNR = 5 dB is used to further investigate the influence of the value of hyperparameters on the model training results, and to provide a reference for improving the training efficiency and recognition effect of the network. The experimental results are shown in Table 6. We can easily find that the appropriate setting of hyperparameters can not only improve the training efficiency of the model, but also improve its identification accuracy to a certain extent. When the epoch value remains unchanged, the recognition accuracy of the model decreases with the increase of the learning rate and the number of convolution kernels. However, with the increase of the batch size, the recognition capability increases. In particular, if the identification effect is not ideal under the current hyperparameter, you can raise the epoch times appropriately. Through repeated training, the training effect of the parameters can be improved.

## 6. Conclusions

In this paper, a micro-motion jamming recognition method based on CV-CNN is proposed. The main advantage of the network is that the complex processing capability is added to the structure of the RV-CNN, so that it is able to calculate the convolution in the complex field directly. At the same time, the echo signal matrix datasets of target echo without jamming and three patterns of micro-motion jamming are constructed, and the input 2-D complex signals are serialized. The simulation results show that CV-CNN can better utilize most of the information of the complex signal and realize more accurate parameter training, thus achieving better recognition results. The simulation results show that when SNR = −5 dB and JSR are in the range of −10 to 20 dB, the recognition accuracy of CV-CNN for micro-motion jamming patterns can reach up to 99.33%, which is nearly 1% better than that of RV-CNN under the same conditions.

## Figures and Tables

**Table 1:** Micro-motion jamming parameters.

Jamming Patterns	Main Parameters	Range of Values
modulation and repeater micro-motion jamming	$r_{P}$ (m)	5~10
	$\omega_{P}$ (rad/s)	10~20
	forwarding times	2~4
micro-motion scattered wave jamming	$r_{P}$ (m)	5~10
	$\omega_{P}$ (rad/s)	10~20
	α (rad)	0~π
	forwarding times	2~4
pulse convolution micro-motion jamming	Δt (ns)	0~90
	time delay interval (ns)	1~5
	time delay range cells	1~7
	forwarding times	2~4

**Table 2:** The structure and parameter settings of RV-CNN.

Layer Name	Main Parameters				
Normalization Layer	Batch Normalization				
**Convolution Layer**	**Conv. Num**	**Conv. Parameters**	**Activation**	**Pooling**	**Dropout**
	Conv. 1	Kernel size: 5 Input num: 1 Output num: 16	ReLU	MaxPooling (2 × 2)	-
	Conv. 2	Kernel size: 7 Input num: 16 Output num: 32	ReLU	MaxPooling (2 × 2)	-
	Conv. 3	Kernel size: 9 Input num: 32 Output num: 64	ReLU	MaxPooling (2 × 2)	50%
**Global Pooling Layer**	Adaptive averaging pooling processing				
**Fully Connected Layer**	Input num: 64 Output num: 4				
**Output Layer**	Softmax				

**Table 3:** The structure and parameter settings of CV-CNN.

Layer Name	Main Parameters				
Normalization Layer	Complex Batch Normalization				
**Convolution Layer**	**Conv. Num**	**Conv. Parameters**	**Activation**	**Pooling**	**Complex Dropout**
	CConv. 1	Kernel size: 5 Input num: 1 Output num: 16	modReLU	CMaxPooling (2 × 2)	-
	CConv. 2	Kernel size: 7 Input num: 16 Output num: 32	modReLU	CMaxPooling (2 × 2)	-
	CConv. 3	Kernel size: 9 Input num: 32 Output num: 64	modReLU	CMaxPooling (2 × 2)	50%
**Global Pooling Layer**	Adaptive averaging pooling processing				
**Complex Fully Connected Layer**	Input num: 64 Output num: 4				
**Output Layer**	Softmax				

**Table 4:** Signal pattern recognition results of different network models when SNR = −5 dB.

Signal Pattern	CV-CNN		RV-CNN	
	Recognition Accuracy (%)	Probability Distance	Recognition Accuracy (%)	Probability Distance
Class1	100	-	100	-
Class2	99.33	12~14.9	98	0.1~0.6
Class3	96.67	1~10	96.67	0.55~0.6
Class4	99.33	4.5~8	96	1.5~1.85

**Table 5:** Effects of different SNRs and different training set sample sizes on the recognition rate of CV-CNN model.

Number of Samples for Each Signal Pattern	Recognition Accuracy (%)		
	SNR = −5 dB	SNR = 0 dB	SNR = 5 dB
N = 50	88.96	96.04	96.89
N = 100	90.28	96.55	97.43
N = 150	91.17	97.36	99
N = 200	95.13	98.28	99.57
N = 250	96.9	98.91	99.85
N = 300	97.37	99.15	99.98

**Table 6:** The influence of main hyperparameter settings on model recognition accuracy.

Hyperparameter	Variation Range	Recognition Accuracy (%)		
		Epoch = 2	Epoch = 5	Epoch = 10
learning rate	[5 × 10^−5^, 5 × 10^−3^]	98.93	99.23	99.86
	[1 × 10^−4^, 1 × 10^−2^]	98.56	98.99	99.23
	[5 × 10^−4^, 5 × 10^−2^]	85.5	95.13	98.87
	[1 × 10^−3^, 1 × 10^−1^]	38.37	68.22	86.57
batch size	BZ = 4	67.5	92.33	96.12
	BZ = 10	81.5	96.37	98.22
	BZ = 15	96.5	97.86	99.51
	BZ = 20	97.5	99.21	99.72
kernel number	(16, 32, 64)	100	100	100
	(32, 64, 128)	99.92	99.86	100
	(64, 128, 256)	99.88	98.67	100
	(128, 256, 512)	98.65	99.12	100

## Data Availability

Not applicable.
